# Endoplasmic reticulum stress remodels alveolar bone formation after tooth extraction

**DOI:** 10.1111/jcmm.15753

**Published:** 2020-09-29

**Authors:** Yun Chen, Yue Guo, Jun Li, Ying‐Yi Chen, Qiong Liu, Li Tan, Zheng‐Rong Gao, Shao‐Hui Zhang, Ying‐Hui Zhou, Yun‐Zhi Feng

**Affiliations:** ^1^ Department of Stomatology The Second Xiangya Hospital Central South University Changsha China; ^2^ Department of Metabolism & Endocrinology National Clinical Research Center for Metabolic Disease The Second Xiangya Hospital Central South University Changsha China

**Keywords:** bone remodelling, endoplasmic reticulum stress, p‐eIF2α, primary calvarial osteoblasts, tooth extraction, unfolded protein response

## Abstract

Bone healing in tooth extraction sockets occurs in a complex environment containing saliva and many microorganisms and is affected by many factors. Endoplasmic reticulum (ER) stress affects bone metabolism, but the role of ER stress in bone healing after tooth extraction remains unclear. We utilized a rat tooth extraction model, in which we promoted wound healing by using salubrinal to regulate the ER stress response. Western blot analysis showed increased expression of p‐eIF2α/eIF2α, Runx2 and alkaline phosphatase (ALP) in bone tissue, and histological assays showed irregularly arranged and new bone with more collagen fibres 14 days after tooth extraction and after modulating the degree of ER stress. Micro‐CT showed that modulating ER stress to an appropriate degree increases bone filling in regards to the density in the bottom and the surrounding bone wall of the tooth extraction wounds. Transmission electron microscopy showed rough ER expansion and newly formed collagen fibrils in osteoblasts after modulating ER stress to an appropriate degree. We also used different concentrations of salubrinal to evaluate the resistance to tunicamycin‐induced ER stress in an osteogenic induction environment. Salubrinal restored the tunicamycin‐induced decrease in the viability of primary calvarial osteoblasts and increased the expression of Runx2 and ALP, and decreased p‐eIF2α/eIF2α in a dose‐dependent manner. Taken together, the results demonstrate that ER stress occurred after tooth extraction, and regulating the degree of ER stress can promote bone healing in tooth extraction sockets, providing clinical evidence for bone healing.

## 1 INTRODUCTION

Tooth extraction is a common dental procedure, and extraction wounds are typically left exposed to the oral cavity, in which many microorganisms reside,^1^ with the involvement of saliva in oral tissue repair processes,^2^ creation of a bony defect, and associated bony and soft tissue changes. Alveolar bone loss occurs after tooth extraction with great individual variation, and bone healing involves many factors,^3^ commonly with mechanical stimulation, ageing, inflammation^4^, ^5^ and bone metabolic disease.^6^ High‐frequency acceleration^7^ or subcutaneously administered PTH^8^ has an osteogenic effect, and can be used to prevent alveolar bone loss and/or accelerate bone healing.

The endoplasmic reticulum (ER) is an important organelle of secreted protein and membrane protein folding.^9^ Ischaemia, hypoxia, massive accumulation of miss folded proteins and an imbalance in calcium ions lead to ER stress.^10^ ER stress is involved in a variety of pathological and physiological processes that are key contributors to a growing list of human diseases.^11^ In vitro observation confirmed that osteoblast differentiation and proliferation increase when ER stress is induced by BMP2,^12^ PTH^13^ and appropriate mechanical stimulation.^14^ In vivo experiments have shown that when the ER stress response is weakened or absent, the ER folding capacity of osteoblasts decreases, leading to a decrease in bone formation capacity, which can be reversed by inducing ER stress.^15^, ^16^ In contrast, ageing, drugs and other effects occurred in cells by inducing ER stress, leading to cell apoptosis, reduced expression of osteogenic markers and decreased osteogenic ability.^17^, ^18^ ER stress induced by alcohol and periodontitis can lead to apoptosis of osteoblasts and bone resorption.^19^, ^20^ In vivo experiments have shown that ER stress plays a critical role in chondrocyte apoptosis and mandibular cartilage thinning in response to compressive mechanical force, which could lead to apoptosis.^21^, ^22^ The effect of ER stress may be influenced by different routes of inducement and duration, resulting in differences in the inhibition or promotion of osteogenesis. Alveolar bone arises from neural crest cells in the neuroectoderm germ layer, which is distinct from the axial and appendicular skeleton of the mesoderm, and undergo intramembranous instead of endochondral ossification.^23^ Despite seeming similar to other bones in the body, alveolar bone serves distinct functions and is the most dynamic tissue in the periodontal system and the skeletal system.^24^ Most research involving ER stress in the study of bone biology has focused on long bones, such as the femur and tibia.^25^, ^26^ Bone healing after tooth extraction involves many factors, but the role of ER stress in the healing process after tooth extraction is unclear. The role of ER stress is dynamically regulated in vivo, and the osteogenic response caused by ER stress is varied in different stages and degrees.^27^, ^28^

When ER stress occurs, it induces the unfolded protein response (UPR), a series of transcriptional and translational events. The UPR can either restore ER homeostasis or activate pro‐apoptotic pathways depending on the type of insult, intensity and duration of the stress, and cell type.^29^, ^30^ The PERK‐eIF2*α*‐ATF4 pathway is one of the key UPR pathways.^9^ During the UPR, phosphorylation of the alpha subunit of eukaryotic initiation factor 2α (eIF2α) is activated and inhibits global protein translation.^31^ The modulation of phosphorylated eIF2α potentially alters the fate of damaged tissues.^28^ Salubrinal has been shown to adapt intracellular stress to protect against ER stress‐induced cell injury ^32^, ^33^ through modulation of eIF2α phosphorylation, promoting the differentiation of osteoblasts both in vivo and vitro.^27^, ^34^

ER stress occurs when the vivo environment changes,^29^ and salubrinal could alter the phosphorylation level of eIF2α, stimulating bone formation.^28^, ^32^ However, the role of ER stress in bone healing after tooth extraction in the complex oral environment remains unclear. The present study aimed to assess the role of ER stress in the bone healing process, and the relationship between the degree of ER stress and bone healing after tooth extraction.

## 2 MATERIALS AND METHODS

### 2.1 Animal preparation

A total of 48 male Sprague‐Dawley rats (age ~8 weeks, body weight 300‐400 g; Hunan SJA Laboratory Animal Co., Ltd) were housed in the Animal Experiment Center of Central South University on a 12:12 hours light‐dark cycle under pathogen‐free conditions and had free access to food and water, and they were allowed to acclimate for 1 week before experimentation. All procedures performed in this study were approved by the Central South University Animal Care and Use Committee and were in compliance with the Guiding Principles in the Care and Use of Animals endorsed by the China Physiological Society. Body weight was used to evaluate general health condition.

### 2.2 Tooth extractions and salubrinal treatment

Experimental tooth extraction was performed on 48 Sprague‐Dawley rats which randomly dived into four groups (n = 12/group): (a) M1 extraction, observation for 7 days; (b) M1 extraction, observation for 14 days; (c) M1 extraction + PBS (3 days post‐extraction), observation for 7 days; (d) M1 extraction + Salubrinal (T3045; Target Mol, resolved in DMSO, a selective eIF2 alpha phosphatase inhibitor which protects cells from ER stress and inhibits apoptosis, 1 mg/kg for 3 days post‐extraction), observation for 7 days. The maxillary M1s were extracted under anaesthesia with intraperitoneally injected 3% pentobarbital sodium (30 mg/kg, with normal saline) and hemostasis by compression, tissue collected from the tooth extraction position. The dorsal skin was used for subcutaneous injection.

### 2.3 Micro‐computed tomography (micro‐CT)

The maxillary alveolar bone was fixed in 4% paraformaldehyde for 48 hours, and then transferred to 70% alcohol at 4°C until use. The interradicular bone of the maxillary M1s was scanned and analysed. The extraction sockets were scanned using a GE eXplore Locus SP Micro‐CT Scanner (GE Healthcare). Image reconstructions and analyses of the mesial roots of the maxillary first molar extraction sockets were segmented by semi‐manual contouring and analysed using Microview and an Advanced Bone Application database (version 2.3; GE Healthcare). Trabecular parameters were identified by the direct‐measures technique.

### 2.4 Histological assays

Maxillary alveolar bone was fixed with 4% paraformaldehyde for 2 days at 4°C and decalcified with 10% ethylenediaminetetraacetic acid (EDTA, pH 7.4) for 21 days. Samples were embedded in paraffin and 4‐μm‐thick sagittal plane slices prepared using an RM2235 microtome (Leica Microsystems GmbH). Masson's trichrome and haematoxylin and eosin (H&E) staining were performed respectively, following the manufacturer's instructions (Beijing Solarbio Science & Technology Co., Ltd.). Alkaline phosphatase (ALP) staining was performed using an ALP assay kit (Sigma‐Aldrich). Sections were scanned on a Scan Scope GL optical microscope (ZEISS, Axiocam 503 color) and collage was quantified by Image J.

### 2.5 Transmission electron microscopy

For transmission electron microscopy, the maxillae were fixed for 48 hours at 4°C in 4% paraformaldehyde and 2.5% glutaraldehyde solution, and the samples decalcified as described above. After fixation, the samples were infiltrated in epoxypropane, and embedded in EPON812. Ultra‐thin 50 nm sections were made using a Leica EM UC7 ultramicrotome (Leica). Sections were counterstained with 3% uranium acetate and lead nitrate and examined using a model 7700 electron microscope (Hitachi) operated at 80 kV. The images of osteoblasts in the extraction wounds were captured with a MEGAVIEW camera.

### 2.6 Primary calvarial osteoblast cultures

Primary calvarial osteoblasts (POBs) were extracted from neonatal (2‐ to 3‐day‐old) C57B6L mice.^35^ POBs were cultured at 37°C in a humidified atmosphere of 5% CO_2_ in α‐MEM with 10% FBS (Gibco, Thermo Fisher) and 1% penicillin‐streptomycin. For differentiation studies, 50 μg/mL of L‐ascorbic acid phosphate (Wako Pure Chemical Industries) and 10 μmol/L beta‐glycerophosphate (Sigma) were added during the medium change. Salubrinal was administered at three different dosages (0.2, 1 and 5 μmol/L) simultaneously with ER stress inducer tunicamycin (Tm; T7765; Sigma; 100 ng/mL) for 48 hours.

For cellular ALP staining, the POBs were quantified utilizing a cell imaging system after 7 days. ALP staining was performed as described above then quantified by Image J.

### 2.7 Cell viability assay

The cell vitality in different degrees of ER stress was assessed using a Cell Counting Kit‐8 (CCK‐8; Beyotime). We administered 10% FBS, 1% penicillin/streptomycin, salubrinal (0.2, 1 or 5 μmol/L) and Tm (100 ng/mL). After 48 hours, the plate was gently rinsed with pre‐warmed PBS before adding 200 μL of reaction medium (50 μL of CCK‐8 solution and 150 μL of medium) to each well. After incubation for 2 hours, the optical density at 450 nm was measured using a spectrophotometer (Bio‐Tek).

### 2.8 Western blot analysis

Bone tissue powders (crushed in liquid nitrogen) from the tooth extraction position and cells were lysed in RIPA lysis buffer, which contained inhibitors of proteases and phosphatases. Western blot was then performed as described previously.^36^ Primary antibody was used at the manufacturer's suggested concentration: eIF2α (1:1000; 11233‐1‐AP; Proteintech), p‐eIF2α (1:1000; Ser51‐D9G8; Cell Signaling), Runx2 (1:1000; ab114133; Abcam) or β‐actin (1:5000; Proteintech). Immunoreactive bands detected by an antirabbit peroxidase‐conjugated secondary antibody (1:5000; Proteintech) and visualized by enhanced chemiluminescence (Amersham Imager 600; General Electric Company). Protein band densitometry was performed using Image J.

### 2.9 Statistical analysis

All data are presented as the means ± standard deviation. All experiments were performed at least three times. Differences between groups were analysed by ANOVA (one‐way or two‐way) or Student's *t* test. A probability of *P* < .05 was considered significant.

## 3 RESULTS

### 3.1 ER stress plays an important role in bone formation and is specifically regulated by salubrinal concentration

**FIGURE 1:**
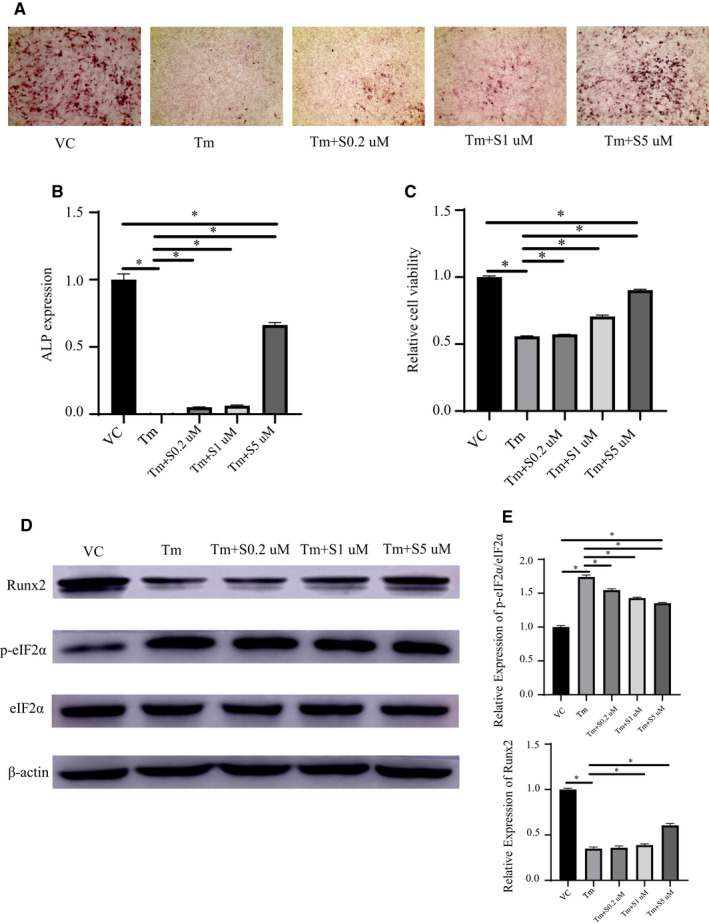
ER stress plays an important role in bone formation and is regulated by salubrinal (SAL) concentration. A‐B, Osteogenic induction in POBs for 7 d with salubrinal administered at 0.2, 1 or 5 μmol/L in the presence of ER stress inducer tunicamycin (Tm; 100 ng/mL). After 48 h, ALP expression (black, 100×) decreased with Tm treatment, which was reversed by SAL. C, CCK8 assay for POBs showing the same trend. D‐E, Representative Western blots showing SAL’s protection of osteogenesis against ER stress. The expression of Runx2/β‐actin protein increased and the expression of p‐eIF2α/eIF2α decreased with recovery of the ER. Data were presented as the ratio with β‐actin and mean ± SD. The asterisks (*) represent *P* < .05. VC, vehicle control

We investigated the changes in ER stress during the induction of osteogenesis in vitro. ALP staining was weakest under the action of Tm; the cells became small and almost no osteogenesis was present compared to the VC group. Under the same conditions, the expression of ALP increased after administration of increasing concentrations of salubrinal (Tm + S0.2/1/5 μmol/L), with ALP staining gradually increasing. ALP staining was not significantly different at low concentrations and reached its highest levels with Tm + S5 μmol/L (Figure 1A,B). Salubrinal restored the Tm‐induced decrease in the viability of POBs in a dose‐dependent manner, but the reverse effect was not obvious at low concentrations of salubrinal (*P* < .05 for Tm + S1 μmol/L and Tm + S5 μmol/L, Figure 1C). Western blot analysis showed Tm increased the level of ER stress‐related markers p‐eIF2α/eIF2α (*P* < .05) but decreased the level of osteogenic marker Runx2 (*P* < .05) in POBs; as the concentration of salubrinal increased, the level of p‐eIF2α/eIF2α (all *P* < .05) decreased, the level of Runx2 increased (all *P* < .05), but the increase in Runx2 expression was not obvious at Tm + S0.2 μmol/L (Figure 1D,E).

### 3.2 ER stress occurred in alveolar bone after tooth extraction

**FIGURE 2:**
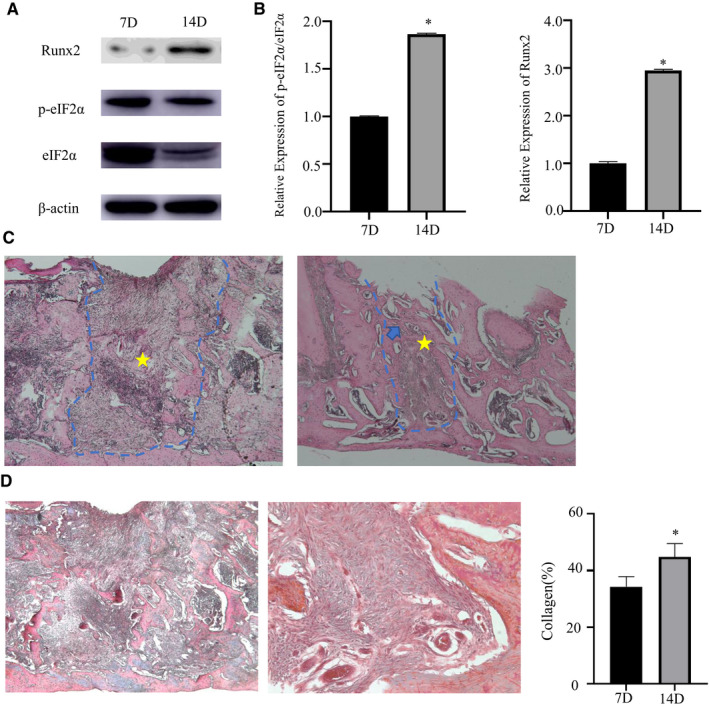
ER stress occurred in alveolar bone after tooth extraction. A, Representative Western blots of the protein expression levels of ER stress‐related proteins eIF2α, p‐eIF2α and osteogenic marker Runx2 in bone tissue at tooth extraction sites 7 and 14 d after tooth extraction. B, p‐eIF2α/eIF2α and Runx2/β‐actin proteins was expressed on days 7 and 14. C‐D, Representative photomicrographs of the stained sections of extraction sockets. The blue dotted line indicates the tooth extraction socket, the star indicates irregular woven bone, and the blue arrow indicates the bone bridge. 14 d significantly increased the number of collagen fibres. Top, HE staining, 50×; bottom, Masson trichrome staining, left 50×, right 200×. Blue‐stained fibres in the connective tissue indicate collagen. Data were presented as the ratio with β‐actin and mean ± SD. The asterisk (*) represents *P* < .05 (n = 12)

In order to observe healing in tooth extraction sockets and the degree of ER stress in different periods, maxillary alveolar bone was collected 7 and 14 days after surgery. Western blot analysis showed that p‐eIF2α/eIF2α and Runx2 are expressed at higher levels at 14 days (both *P* < .05, Figure 2A,B). At 7 days, woven bone (Figure 2C, star) formation occurred on the developing trabeculae. At 14 days, the bone wall was absorbed, and the temporary matrix formed direct communication with the surrounding cancellous bone. The irregular woven bone and calcified bone tissue were formed in the tooth extraction socket. The bone bridge (Figure 2C, blue arrow) was connected at the top to form the preliminary osseous seal of the alveolar socket. By 14 days, the collagen content in connective tissue was significantly increased. No cartilage formation was observed in either group (Figure 2D).

### 3.3 Regulating the degree of ER stress could accelerate bone formation

**FIGURE 3:**
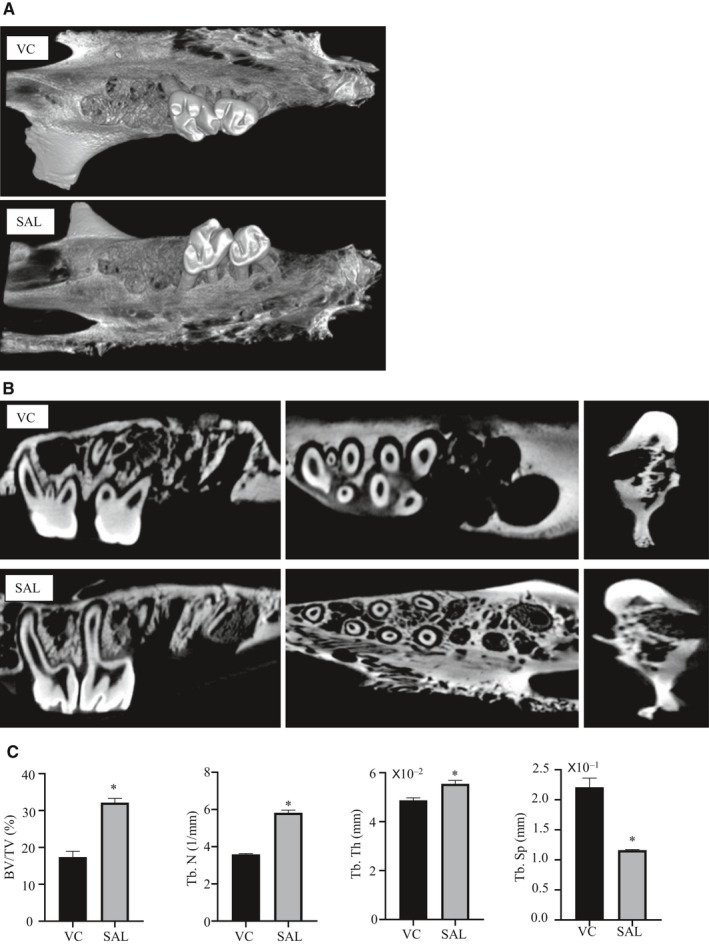
Suppressing ER stress could accelerate bone formation. A, Three‐dimensional micro‐CT reconstruction images of the vehicle control (VC) and salubrinal (SAL) groups. B, In the first molar area, bone formation was greater in the SAL group in the sagittal view (alveolar ridge median sagittal plane), occlusal view (root bifurcation surface) and coronal view (maxillary first molar mesial root). C, Micro‐CT assessment of the mesial roots of the maxillary first molars extraction sockets. SAL group BV/TV, Tb.N and Tb.Th were all increased, but Tb.Sp was decreased compared with the VC group. Data were presented as mean ± SD. The asterisks (*) represent *P* < .05 (n = 3)

To investigate the regulatory function of suppressing ER stress on bone remodelling after tooth extraction, we performed micro‐CT on the maxillary alveolar bone of rats with and without salubrinal injection 7 days after tooth extraction. In the 3D reconstruction, qualitative assessment of the first molar extraction site indicated a marked increase in bone filling in the salubrinal group compared to the VC group (Figure 3A). From the three different views, it was clear that the height and the width of the alveolar bone in the salubrinal group decreased less than in the VC group, and the interroot bone was well preserved. Lower density new bone formation was present in the bottom of the tooth extraction socket and the surrounding bone wall in the salubrinal group (Figure 3B). The salubrinal group expressed a significant elevation in the total volume (BV/TV), the trabecular number (Tb.N) and the trabecular thickness (Tb.Th), together with a significant reduction in the trabecular separation(Tb.Sp), compared to the VC group (Figure 3C).

### 3.4 Regulating the degree of ER stress could protect osteogenesis in vivo

**FIGURE 4:**
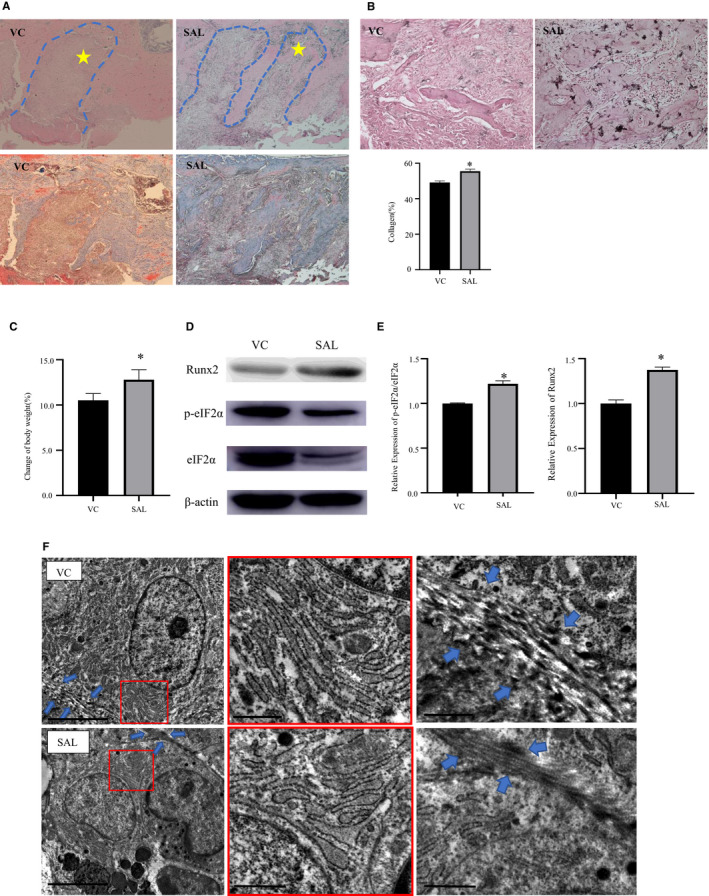
Suppressing ER stress could protect osteogenesis in vivo. A, Representative photomicrographs of tooth extraction sockets in the salubrinal (SAL) and vehicle control (VC) groups. The blue dotted line indicates the tooth extraction socket, and the star indicates irregular woven bone. SAL group significantly increased the number of collagen fibres. Top: HE staining, 50×; bottom: Masson's tricolour staining, 50×, blue fibres in connective tissue represent collagen. B, Representative photomicrographs of ALP staining (200×) in tooth extraction sockets in the SAL and control groups. C, The percentage change in body weight. Body weight increased after using salubrinal. D‐E, Representative Western blots of the protein expression levels of eIF2α, p‐eIF2α and Runx2 in the bone tissue around the tooth extraction wounds with different drug interventions. p‐eIF2α/eIF2α and Runx2/β‐actin protein expression was higher after salubrinal use. F, Transmission electron microscope images showing the ultrastructural changes of the rough ER (red square) and the formation of collagen fibres (blue arrows) in osteoblast around the sockets in the SAL and control groups (top: PBS, bottom: SAL) Left scale bar = 5 μm, middle and right scale bar = 1 μm. Data were presented as the ratio with β‐actin and mean ± SD. The asterisks (*) represent *P* < .05(n = 9)

To further identify the mechanism of bone formation after tooth extraction by suppressing ER stress, we analysed the tooth extraction sockets of bone specimens 7 days after tooth extraction. After using salubrinal, the central area of the tooth extraction socket had woven bone (Figure 4A, star) formation, and the new bone was arranged irregularly, with bone matrix deposition in the bottom and lateral wall (Figure 4A, blue dotted line). The salubrinal group had more collagen expression in the tooth extraction socket (Figure 4A). The ALP staining was higher in the salubrinal group than the VC group (Figure 4B). No infections were detected at the injection sites during the processes, and we did not observe any abnormal behaviour or changes in food intake. During the 7‐day experiment, both groups of rats had increased body weight, with a greater increase in the salubrinal group than the VC group (*P* < .05, Figure 4C). Western blot analysis of the bone tissue from the tooth extraction sites showed that p‐eIF2α/eIF2α and Runx2 were expressed at higher levels in the salubrinal group (both *P* < .05, Figure 4D,E). Osteoblasts in the maxillary alveolar bone were analysed by electron microscopy to characterize the ER morphology. Compared to the VC group, the salubrinal group presented a significant increase in the percentage of rough ER area (Figure 4F, red square) and newly formed collagen fibrils (Figure 4F, blue arrows), with a striated pattern lined up parallel to the cell surface and extending along its elongated cytoplasm to its neighbouring cells in osteoblasts. Collagen fibre formation is not obvious in the VC group.

## 4 DISCUSSION

This study showed that ER stress occurs after tooth extraction and significantly correlates with alveolar bone resorption. The phosphorylation of eIF2α varied at different times after tooth extraction and was associated with bone formation. Using a rat tooth extraction model, we found that adjusting intracellular stress to protect against ER stress‐induced cell injury by modulation of the phosphorylation of eIF2α has a positive effect on tooth extraction socket healing and could improve bone formation after tooth extraction.

**FIGURE 5:**
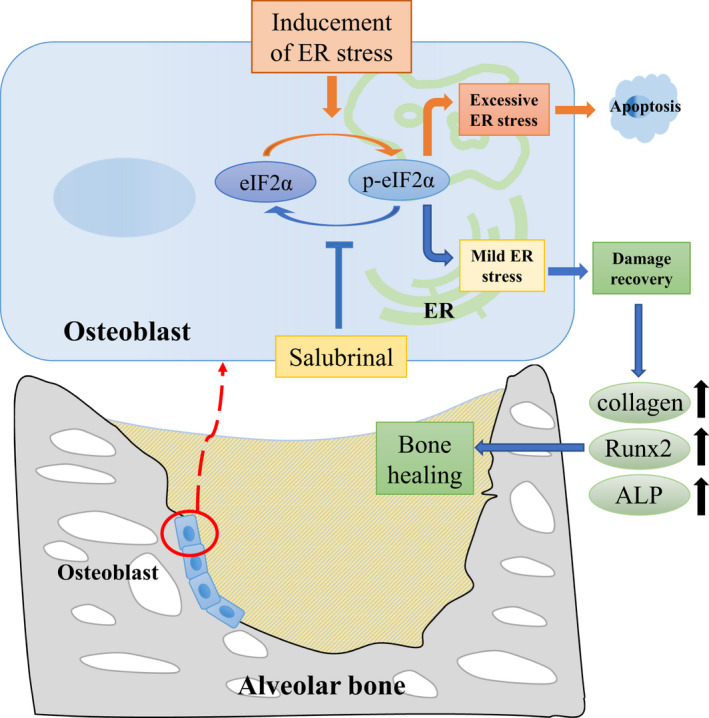
Proposed mechanism of action. ER stress occurred in the alveolar bone after tooth extraction, and excessive ER stress can cause osteoblast apoptosis. Salubrinal promoted osteoblastogenesis and bone healing through regulation of eIF2α

We found that different degrees of ER stress can affect the osteogenic differentiation of POBs. This finding is consistent with a previous study reporting that specific inhibition of ER stress blocked apoptosis and decreased mineralization induced by either glucocorticoid or ER stress inducer Tm in OB‐6 cells or POBs.^18^ ALP and Runx2 expression were weakest under the action of Tm, but p‐eIF2α/eIF2α expression was highest in this context; thus, the increase in the basal p‐eIF2α level by Tm indicates that the treated cells undergo some sort of stress,^37^ and excessive ER stress inhibits osteogenic differentiation and causes apoptosis^38^ (Figure 5). As the concentration of salubrinal increased, the level of p‐eIF2α/eIF2α decreased, but the levels of p‐eIF2α/eIF2α have previously been shown to increase 48 hours after administration of 100 ng/mL Tm with 5 μmol/L salubrinal in MC3T3‐E1 cells.^26^, ^27^ The differences may be related to the use of different types of cells. Therefore, reducing ER stress is conducive to cell differentiation and proliferation, and controlling ER stress at a reduced level would promote osteogenesis. The reverse effect is not obvious at low concentration of salubrinal, which means that the cells were still in a high degree of ER stress and apoptosis increased. Maintaining p‐eIF2α levels above a certain threshold during stress appears to be critical to ensure post‐stress survival, whereas early recovery decreases viability.^15^, ^39^

Wound healing is a dynamic process.^2^ We used the rat tooth extraction model to explore the relationship between ER stress and different bone healing stages. ER stress‐related marker p‐eIF2α/eIF2α and osteogenic marker Runx2 expression was higher 14 days after tooth extraction. The UPR was already activated on day 14 to promote bone formation and wound healing through the PERK‐eIF2*α*‐ATF4 pathway. Histological examination by H&E and Masson's trichrome staining showed marked bone formation activity already occurred in the extraction socket tissue 7 days post‐tooth extraction with woven bone formation and collagen secretion. This period coincides with the stage in which there is a predominance of bone trabeculae neoformation, with marked osteoblastic activity on the new trabeculae. For the alveolar bone, blood is abundant, bone remodelling is faster, and 7 days, which is just the stage of wound healing that the tooth extraction sockets begin the stage of bone formation.

Micro‐CT analyses showed a marked increase in bone filling in the salubrinal group compared to the VC group, and there was an obvious increase in density in the tooth extraction wounds, suggesting bone formation. Histological examination showed irregularly arranged and new bone with more collagen fibres and the ALP staining was higher than the control group, which is consistent with local administration of salubrinal to inhibit ER stress and accelerate healing as shown with surgically induced bone holes in rat femurs.^28^ In the model of ischaemic osteonecrosis of the femoral head, salubrinal improved symptoms by enhancing angiogenesis and bone healing via modulation of ER stress.^26^ Modulating the phosphorylated level of eIF2α can promote osteogenesis and accelerate healing in vivo by activating the UPR through the PERK‐eIF2*α*‐ATF4 pathway (Figure 5). In addition, salubrinal can also promote osteogenesis and reduce osteoclast through the indirect effect on fibroblast and myoblasts, and then accelerate bone healing.^40^, ^41^ After using salubrinal, the levels of p‐eIF2α/eIF2α increased, confirming that the dephosphorylation of eIF2α was blocked, initiating the UPR reaction and adjusting intracellular stress, which plays a critical role in the responses to ER stress‐induced cell injury but is inconsistent with the results of in vitro experiments. After using salubrinal, the levels of p‐eIF2α/eIF2α decreased, which may be related to the diversity of cell types and complex environment in tooth extraction wounds. We also observed, based on weight gain, that salubrinal is safe, but there is a possibility of systemic side effects.

Transmission electron microscopy showed that the osteoblasts around wounds had a significant increase in the percentage of rough ER area. This is not consistent with the recovery of the ER after using salubrinal, but that cells were under other stimulating conditions, such as osteoblasts from the distal femur of mice with disuse osteoporosis and chondrocytes from the mandibular cartilage under compressive mechanical stress in rats.^22^, ^27^ The findings may be related to the complex oral environment and the unique structure and function of alveolar bone,^24^ resulting in slower ER recovery, and we observed newly formed collagen fibrils parallel to the cell surface of osteoblasts in tooth extraction wounds after injecting salubrinal, which revealed that mild ER stress could promote osteogenic differentiation. Type I collagen has been reported to enhance osteoblastic differentiation and bone formation.^42^ Our previous study^43^ found that type I collagen plays an important role in the regulation of osteogenesis via Irs‐1/miR‐342 and leads to increased osteogenesis. These previous results are supported by osteoblast differentiation into mature osteoblasts with collagen fibril synthesis.

We demonstrated that osteoblasts could restore their proliferation and osteogenic ability when salubrinal is used to inhibit ER stress and verified that ER stress occurs after tooth extraction. Regulating the degree of the ER stress response can promote bone healing in tooth extraction sockets, but it requires further study.

## CONFLICT OF INTEREST

The authors declare that there is no conflict of interest regarding the publication of this article.

## AUTHOR CONTRIBUTIONS

**Yun Chen:** Investigation (equal); Writing‐original draft (equal); Writing‐review & editing (equal). **Yue Guo:** Funding acquisition (supporting); Investigation (equal); Methodology (supporting); Visualization (equal); Writing‐original draft (equal); Writing‐review & editing (equal). **Jun Li:** Investigation (supporting). **Ying‐Yi Chen:** Investigation (supporting). **Qiong Liu:** Investigation (supporting). **Li Tan:** Investigation (supporting). **Zheng‐Rong Gao:** Investigation (supporting). **Shao‐Hui Zhang:** Investigation (supporting). **Ying‐Hui Zhou:** Methodology (supporting). **Yun‐Zhi Feng:** Conceptualization (lead); Data curation (lead); Formal analysis (lead); Funding acquisition (lead); Investigation (lead); Methodology (lead); Project administration (lead); Supervision (lead); Validation (lead); Visualization (lead); Writing‐original draft (lead); Writing‐review & editing (lead).

## Data Availability

The data used to support the findings of this study are included within the article.
